# Motivating cascade testing for familial hypercholesterolemia: applying the extended parallel process model for clinician communication

**DOI:** 10.1093/tbm/ibac018

**Published:** 2022-04-16

**Authors:** Gemme Campbell-Salome, Nicole L Walters, Ilene G Ladd, Amanda Sheldon, Catherine Davis Ahmed, Andrew Brangan, Megan N McMinn, Alanna K Rahm, Marci L B Schwartz, Eric Tricou, Carla L Fisher, Amy C Sturm

**Affiliations:** Genomic Medicine Institute, Geisinger, Danville, PA, USA; College of Journalism and Communications, University of Florida, Gainesville, FL, USA; Genomic Medicine Institute, Geisinger, Danville, PA, USA; Genomic Medicine Institute, Geisinger, Danville, PA, USA; The FH Foundation, Pasadena, CA, USA; The FH Foundation, Pasadena, CA, USA; Genomic Medicine Institute, Geisinger, Danville, PA, USA; Genomic Medicine Institute, Geisinger, Danville, PA, USA; Genomic Medicine Institute, Geisinger, Danville, PA, USA; Genomic Medicine Institute, Geisinger, Danville, PA, USA; Genomic Medicine Institute, Geisinger, Danville, PA, USA; College of Journalism and Communications, University of Florida, Gainesville, FL, USA; Genomic Medicine Institute, Geisinger, Danville, PA, USA; Heart Institute, Geisinger, Danville, PA,USA

**Keywords:** Risk communication, Genetic testing, Cascade testing, Familial hypercholesterolemia, Health communication, extended parallel process model

## Abstract

Motivating at-risk relatives to undergo cascade testing for familial hypercholesterolemia (FH) is critical for diagnosis and lifesaving treatment. As credible sources of information, clinicians can assist in family communication about FH and motivate cascade testing uptake. However, there are no guidelines regarding how clinicians should effectively communicate with probands (the first person diagnosed in the family) and at-risk relatives. Individuals and families with FH can inform our understanding of the most effective communications to promote cascade testing. Guided by the extended parallel process model (EPPM), we analyzed the perspectives of individuals and families with FH for effective messaging clinicians can use to promote cascade testing uptake. We analyzed narrative data from interviews and surveys collected as part of a larger mixed-methods study. The EPPM was used to identify message features recommended by individuals and families with FH that focus on four key constructs (severity, susceptibility, response efficacy, self-efficacy) to promote cascade testing. Participants included 22 individuals from 11 dyadic interviews and 98 survey respondents. Participants described prioritizing multiple messages that address each EPPM construct to alert relatives about their risk. They illustrated strategies clinicians could use within each EPPM construct to communicate to at-risk relatives about the importance of pursuing diagnosis via cascade testing and subsequent treatment for high cholesterol due to FH. Findings provide guidance on effective messaging to motivate cascade testing uptake for FH and demonstrates how the EPPM may guide communication with at-risk relatives about genetic risk and motivate cascade testing broadly.

## Teaser

Individuals and families with familial hypercholesterolemia (FH) suggested clinicians use a combination of fear and efficacy messages to motivate at-risk relatives to pursue cascade testing.

**Implications**

**Practice:** Clinicians can follow the recommendations of individuals and families with FH and use their examples to design risk communication messages about FH that include fear and efficacy appeals to motivate cascade testing and potentially treatment adherence.

**Policy:** Policymakers need to prioritize FH identification by implementing cascade testing programs nationally and addressing barriers by reducing the cost of genetic testing, protections against health and life insurance discrimination for individuals and families with FH, and supporting the expansion of clinical genetics services to improve access to testing.

**Research:** Future research should be aimed at testing message effects based on participants’ recommendations and EPPM constructs delivered by clinicians in the context of FH and other heritable cardiovascular disease and cancer contexts.

## Introduction

Familial hypercholesterolemia (FH) is a common genetic disorder associated with increased risk of premature atherosclerotic cardiovascular disease due to lifelong exposure to high low-density lipoprotein cholesterol (LDL-C) [1, 2]. If left untreated, individuals with FH have up to a 20-fold increased risk for cardiovascular disease compared with the general population [3]. However, when FH is diagnosed and treated early in life, this risk is significantly reduced [4, 5]. Although early diagnosis and treatment can be lifesaving, estimates show 90% or 1.1 million of the 1 in 250 people in the USA with FH remain undiagnosed [6]. When diagnosis is made for FH, it often comes decades too late to prevent heart attacks and early death [7]. The identification of individuals with FH through cascade testing has the Centers for Disease Control and Prevention’s (CDC) Tier 1 evidence for reducing morbidity and mortality [8].

Individuals with FH can be identified through clinical methods such as collecting a family health history and completing a blood test to check cholesterol levels as well as through genetic testing for pathogenic variants in the main three genes associated with FH, *LDLR*, *APOB*, and *PCSK9* [1, 3]. Most individuals with FH have an autosomal dominant form of the condition, so cascade testing, or systematically screening at-risk relatives of individuals with FH, is a particularly effective method of identifying additional individuals with FH [9, 10]. Previous research demonstrates that when individuals with FH are diagnosed and their at-risk relatives are tested, preventive interventions and treatments can begin at a young age and improve health outcomes [10]. However, in the USA, FH identification and cascade testing are not systematically performed and the burden is placed on the proband, or first person diagnosed with FH in the family, to communicate with and motivate at-risk relatives to pursue cascade testing [11].

### Clinicians’ role in motivating cascade testing

Probands have reported challenges recalling and communicating complex risk information about FH with relatives and have expressed interest in receiving support from clinicians to assist in their family communication and motivating their relatives to pursue cascade testing [12–14]. At-risk relatives may perceive clinicians as credible and authoritative sources of information, which can better motivate at-risk relatives to pursue cascade testing [15, 16]. Previous research has shown that communication methods from clinicians such as “Dear Family” letters [17], digital tools like chatbots [18], and direct contact programs [10, 19] are acceptable to patients, families, and clinicians, and can be effective for identifying more individuals with FH [11, 19, 20].

While clinicians may be well-situated to support family communication and cascade testing, there are no universally accepted guidelines regarding how to best communicate with the proband and family members to enhance understanding and motivate cascade testing across genetic conditions [21, 22]. Even when the diagnostic criteria for FH are known and met, FH diagnosis is often delayed or missed, which may be partly due to ineffective clinical communication of the diagnosis and its implications for families [7]. Individuals with FH are often aware that they have high cholesterol but may not understand or be told that their high cholesterol is genetic and due to FH, how serious FH is, and/or that FH requires early medical intervention [23]. Little research has focused on what messages from clinicians inform probands and at-risk relatives of their potential hereditary disease risks and motivate them to pursue cascade testing. Further, investigating how to communicate about risk for FH and what messages resonate with individuals can guide clinicians on how to effectively motivate at-risk relatives to pursue cascade testing and adhere to evidence-based interventions for FH.

### Theoretically guided messaging about cascade testing

When individuals face disease risk, they encounter fear and evaluate that fear to determine how to respond. The extended parallel process model (EPPM) is a validated, contemporary model for understanding how individuals evaluate and respond to health threats and guides risk communication to promote health protective behaviors [24, 25]. The EPPM has been applied to messages promoting health protective behaviors in contexts of smoking cessation, sexual health, heart disease, and cancer screening [26–28]. The EPPM incorporates fear as a central construct in risk communication for motivating behavior change and highlights the importance of incorporating efficacy in messages with fear appeals [25]. The EPPM provides a useful lens to examine how clinicians can effectively communicate to motivate cascade testing for FH.

The EPPM posits that individuals will take a recommended action based on two phases of appraisal in response to messages containing fear appeals [24]. When a clinician communicates with a relative that they are at risk for FH, that message inherently contains a fear appeal and initiates the first phase, in which the relative evaluates the perceived threat of having FH. Perceived threat is evaluated based on an individuals’ appraisal of *perceived severity* (i.e., how serious are the health risks related to FH?) and *perceived susceptibility* (i.e., how likely am I to face health risks related to FH?). The more individuals believe they are susceptible to a serious health threat, the more likely they are to move to the second appraisal phase [25]. If at-risk relatives perceive the threat of FH to be irrelevant, they may discontinue message processing with the clinician (e.g., stop communicating, tune out, etc.) and will not be motivated to evaluate their efficacy to pursue cascade testing.

In the second appraisal phase, the fear experienced increases motivation to assess their efficacy or an individual’s perceived ability to respond to the health threat or, in this context, their efficacy to pursue cascade testing. Perceived efficacy consists of *response efficacy* or how effective the individual feels the recommended behavior will be in managing the threat (i.e., how effective is cascade testing in determining FH risk?) and *self-efficacy*, or the assessment of the individual’s ability to perform the recommended behavior (i.e., am I capable of pursuing cascade testing?) [25]. EPPM research contends that when perceived threat and efficacy are high, individuals engage in a *danger control response* by doing the recommended action. If perceived efficacy is low and perceived threat is high, individuals will engage in a *fear control response* and cope with the increased fear by avoiding, denying, or reacting against the message [24]. This study engaged individuals and families with FH to determine the specific fear and efficacy appeals relevant for families with a history of FH. We posit two inquiries guided by the EPPM:

1. : What message features attending to fear (severity and susceptibility) do individuals and families with FH describe as important for motivating at-risk relatives to engage in FH cascade testing?
2. : What message features attending to efficacy (response efficacy and self-efficacy) do individuals and families with FH describe as important for motivating at-risk relatives to engage in FH cascade testing?

## METHODS

### Study design and participants

The current study involved multiple methods (interviews and surveys) to capture narrative data drawn from a larger mixed-method study aimed at refining health care messages and communication tools to improve FH cascade testing uptake (for a detailed description of the larger study, see [29]). A combination of purposive and snowball sampling was used to recruit participants to capture the breadth and depth of feedback needed for messages that will reach these individuals and families with FH collectively. Eligible participants had to be (a) diagnosed with FH through genetic testing or other clinical methods, (b) an at-risk family member, and/or (c) a family member of someone with FH [30]. This sampling method ensured the study team could capture different perspectives among the intended recipients of messages from clinicians and better illustrate family dynamics that could be important to effective message design.

Dyadic family interviews were conducted to capture feedback on communication tools and allowed researchers to examine differences in communication preferences among family members. The survey was designed to capture the breadth of feedback among individuals with FH and their relatives. Each method presented current communication tools (i.e., letter, chatbot) offered to individuals with FH and at-risk relatives, a description of a potential direct contact program, and a description of cascade testing options. Participants reviewed these materials and provided feedback to (re)design each communication tool to better motivate relatives to pursue cascade testing for FH.

### Procedures

Participants included in this analysis were recruited from Geisinger’s MyCode Community Health Initiative (MyCode), Geisinger’s Multidisciplinary Lipid Clinic (MDLC), and via the FH Foundation. MyCode is a population-based precision health project that combines electronic health record data with genomic data generated from exome sequencing as part of a genomic screening initiative to return clinically actionable results to patient-participants [31, 32]. The FH Foundation is a patient-centered research and advocacy organization dedicated to improving FH identification and care. Participants were invited to either participate in a dyadic interview or respond to a survey. Both study methods focused on gathering participants’ feedback on how to optimize communication materials to support family communication about FH, improve uptake of cascade testing among at-risk relatives, and design messages from clinicians. Participants who completed interviews received a $20 Amazon gift card for their participation. Survey participants recruited from Geisinger were entered into a raffle to win one of five $50 Amazon gift cards.

For dyadic interviews, the participant with an FH diagnosis was invited to the study and asked to recruit a family member to join their interview. Interviews were conducted by phone and audio-recorded, lasting 45–60 min and resulting in over 200 transcribed pages. Transcripts were de-identified, checked for accuracy, and analyzed by the study team. Open-ended survey responses were exported from the survey, de-identified, and checked for accuracy by ensuring there was only one response per IP address, before their inclusion in the full dataset.

### Data analysis

The first two authors (G.C.S, N.L.W) conducted the interviews and debriefed after each interview to discuss emergent patterns, refine probes, and ensure saturation [33]. Interviews were thematically analyzed in conjunction with the open-ended survey data using the constant comparative method and sensitizing constructs from EPPM [34]. The study team (G.C.S., N.L.W., I.L., A.S., A.C.S.) first independently open coded three interview transcripts to confirm participants’ reports were informed by EPPM constructs [24]. A codebook was developed to define and operationalize each sensitizing construct (severity, susceptibility, response efficacy, self-efficacy). Each coder segmented the data according to each construct. Team meetings were held to collapse segmented data prior to beginning a thematic analysis. Open coding was then conducted to identify message features addressing each research question. This included identifying concepts and assigning codes to emergent patterns and then collapsing categories to identify emergent themes [35]. Team meetings were held across the analytical process to collapse analyses, address inconsistencies, and refine the codebook. To ensure rich description, memos were kept for each theme informing each EPPM construct.

## RESULTS

### Participant characteristics

In total, 120 participants are included in this study (see Table 1). Eleven family dyads (*n* = 22 individuals) were interviewed between July and August 2020 and identified as Caucasian and Non-Hispanic/Latino. Separately, 98 participants responded to surveys conducted August–September 2020.

**Table 1:** Participant characteristics

Dyadic interviews	*N* = 22	
Sex	Male (22.7%)	Female (77.3%)
Age ranges	25–34 (18.2%) 45−54 (22.7%) >65 (31.8%)	35–44 (13.6%) 55–64 (13.6%)
Dyadic relationships	Sisters (*N* = 3) Mother–daughter (*N* = 3) Father–daughter (*N* = 1)	Spouse (*N* = 2) Mother–Son (*N* = 2)
Education	High school/GED (22.7%) Trade/technical degree (4.5%) Postgraduate degree (22.7%)	Some College (22.7%) College graduate (27.3%)
Household income	$15−30,000 (4.5%) $75−100,000 (9.1%) $150−200,000 (9.1%) Prefer not to answer (40.9%)	$50−75,000 (13.6%) $100−150,000 (18.2%) $>200,000 (4.5%)
Working for pay	Yes (50%)	No (50%)
FH diagnosis/risk status	Diagnosed (68.2%) Spouse/caregiver (9.1%)	At risk (22.7%)
Insurance status	Private (86.4%) Medicare (31.8%)	Medicaid (4.5%) Tricare/Military (9.1%)
Survey responses	*N* = 98	
Participant type	Individual with FH from Geisinger (*n* = 19, 19.4%) Individual with FH from FH Foundation (*n* = 72, 73.5%) Family member of an individual with FH (*n* = 7, 7.1%)	
Sex	Male (25.5%)	Female (74.5%)
Age	14–80 (*M* = 55.94, *SD* = 13.45)	
Education	Some high school (2%) Some college (13.3%) Bachelor’s degree (36.7%) Prefer not to answer (1%)	High school/GED (10.2%) Associate degree (8.2%) Postgraduate degree (28.6%)
Household income	<$25,000 (6.1%) $50−75,000 (10.2%) Prefer not to answer (18.4%)	$25−50,000 (7.1%) $75−100,000 (18.4%) >$100,000 (39.8%)
FH diagnosis/risk status	Diagnosed (95.9%) Caregiver (3.1%)	At risk (1%)
Diagnostic journey^a^	MyCode result (12.2%) Doctor diagnosed (60.2%)	Genetic test (19.4%) Family history (11.2%)

*FH* familial hypercholesterolemia.

^a^Diagnostic journey refers to the way in which a participant learned that they have/likely have FH. In responding to this survey item, participants could choose more than one of the options listed in the table as contributing to their diagnosis for FH.

### Results overview

**Fig. 1:**
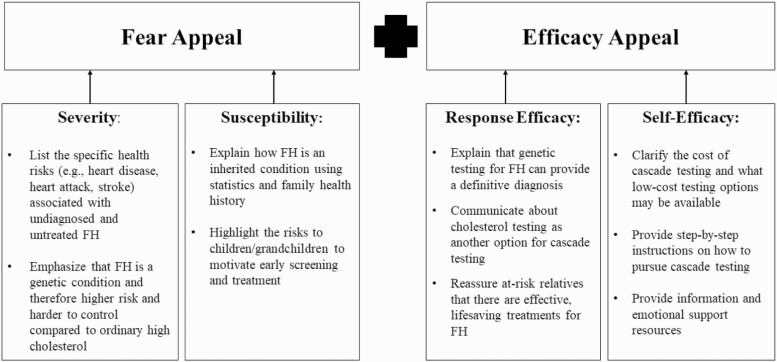
Key themes for applying the EPPM to clinicians’ risk communication.

Participants provided feedback on communication materials, described the persuasive messages they employ with at-risk relatives, and recalled effective messages their clinicians used with them that could be communicated to alert relatives about their risk and overcome their complacency or avoidance. Participants suggested message features clinicians could use to communicate about the importance of pursuing diagnosis for FH, which were consistent with the EPPM constructs of severity, susceptibility, response efficacy, and self-efficacy (see Fig. 1). Exemplar quotes indicate method type with transcript number [dyadic interview (DI1) or survey (S1)] and type of participant: individual diagnosed with FH (FH-Dx), a family member at-risk for FH (FM-AR), or a family member who was not at-risk for FH (FM-NAR).

### RQ1 (fear): attending to severity

Participants suggested two message features to address their relatives’ fear and the construct of severity to ensure their loved ones took their risk of FH seriously. First, participants prioritized the message of *listing the specific health risks associated with undiagnosed/untreated FH*. They specifically suggested clinicians mention risks of heart disease, heart attack, stroke, and early death. Participants also recommended clinicians explain to relatives the risks of lifelong exposure to high cholesterol due to FH, noting: “It’s that lifelong exposure [to high cholesterol] that causes the issues” (FM-AR, DI3). Participants also provided specific language suggestions for clinicians to better address severity. For instance, a participant diagnosed with FH stated: “include ‘if not treated, FH may cause early heart disease, stroke or death.’ Although death is scary or alarming, it might cause someone to take it even more seriously, and take action” (FH-Dx, S26). Another participant reiterated this, saying, “The fact that it puts me at 20 times more at risk for heart attack if I’m not taking care—hearing those statistics are important, but doing it in such a way that you are not scaring people” (FH-Dx, DI6). Participants were careful to explain how these fear appeals should alarm rather than terrify family members and explained that they believed this strategy would get their relatives’ attention and motivate them to continue reading materials or talking with a clinician and pursue screening because, “you only have one heart” (FH-Dx, DI10).

Second, participants advised *emphasizing that FH is a genetic condition and therefore higher risk and harder to control compared to ordinary high cholesterol.* For instance, one participant suggested explaining that “this [genetic variant for FH] increases risk for early heart attack and stroke. … It’s an inherited thing and likely not going to be able to get [your cholesterol] down on your own because [of] it being an inherited diagnosis” (FH-Dx, DI5). Participants recommended distinguishing FH as a serious genetic condition, requiring medical intervention, unlike ordinary cholesterol related to lifestyle factors.

### RQ1 (fear): explaining susceptibility

Participants identified two message features to address their relatives’ fear and construct of susceptibility or their likelihood of having FH. First, participants advised *explaining how FH is an inherited condition using statistics and family health history*. Participants said clinicians should explain how FH is a genetic condition to heighten relatives’ sense of susceptibility by explaining, “These are loved ones and are at a 50% risk of having the genetic defect” (FH-DX, S14). They specifically suggested that clinicians use risk percentages:

Explaining the 50% of the genetic component there, that my boys have a 50% chance of having [FH]. One of my parents probably has this. That makes my brother have a 50% chance. I mean, hearing those statistics were important to me. (FH-Dx, DI6)

Several participants also advised clinicians to bring up past health events in the family related to FH to help relatives understand their susceptibility and motivate testing for FH. For instance, this participant explained:

If you have heart attacks in the family or strokes, I mean that could grab their attention if they had a relative or somebody that they know of that died, then they’ll be like, “Oh, well maybe that’s why they died, and I should get tested.” (FH-Dx, DI8)

Second, participants recommended *highlighting the risks to children/grandchildren to motivate early screening and treatment*. They reported that by stressing the potential risk (susceptibility) to children and/or grandchildren, it would better motivate relatives to take a family-focused approach to testing. Participants indicated clinicians could motivate their relatives by focusing on future generations’ susceptibility rather than just protecting their own health. This participant suggested saying, *“*[Testing] is something to consider, if not even for yourself, for your children and your grandchildren, to open up your mind to it. It could actually help a couple of generations” (FH-Dx, DI4). Another participant described testing as helping “map the family tree and help others in our family” (FH-DX, S68).

In addition, participants stated that it was important for their relatives to understand that FH can impact younger generations’ health now (as opposed to only being a concern later in life), which further appealed to addressing the susceptibility of younger relatives. Participants recommended that clinicians could reframe fear appeals from an individual focus to a family focus by highlighting the FH-related health risks and susceptibility of children. For example, this participant explained this further:

There’s a lot of young kids who have it. That is incredibly frightening to me. They’re on statins at a very young age because they’re showing symptoms that young. And I think it’s imperative that people need to know that their younger kids need to be checked also. … But sometimes people don’t always do stuff for themselves, but when they hear it could affect their own kids… (FH-Dx, DI6)

Some participants also recommended comparing FH screening and treatment to diabetes or cancer screening and treatment to motivate relatives to determine FH risks for their children:

You do feel a sense of guilt if you have passed it on to your child. But you have to be realistic about it. This is a risk factor. It’s almost like Type 1 diabetes. You want to know so that you can hopefully keep that child from experiencing things that you’re experiencing. (FH-Dx, DI1)

### RQ2 (efficacy): improving response efficacy

Participants identified three message features to address response efficacy or their perception of how effective the recommended behavior would be in determining FH risk. First, participants suggested clinicians prioritize *explaining that genetic testing for FH can provide a definitive diagnosis.* They recommended explaining to at-risk relatives that they may not have FH and testing could diagnose or rule out FH when the proband has been genetically identified. For instance, as this participant stated, “Let them know that, just because they’re getting the test, doesn’t mean that they’ll have [FH]” (FH-Dx, DI1). Participants recommended stressing “the importance of getting tested, [it] could save your life” (FH-DX, S68). Participants said it was important to clarify that this test not only provided answers and, thus, was “effective” but was also “quick” and “simple.” Moreover, they suggested explaining how results from testing would give relatives an idea of *“*how urgently they need to act on [FH]” (FH-Dx, S27) to “do some type of intervention to stop or slow down the risk” (FM-AR, DI7).

Second, participants recommended *communicating about cholesterol testing as another option for cascade testing*. After reviewing the study communication materials and descriptions of genetic testing options, participants stated that they wanted more information about cholesterol testing. Some participants perceived the materials as too heavily focused on genetic testing and suggested a balanced description of cholesterol testing as a form of cascade testing:

[The materials] seemed to be leading me that you should get genetic testing, and I would probably want to explore my options and go to my doctor and ask if he thought that was the right move or just regular cholesterol testing. At least arm me with the information I need, and if I felt like I was comfortable, maybe I would schedule an appointment. (FM-AR, DI6)

Participants indicated that this was important prior to testing, as they “would want a personalized discussion of the risks and benefits of adding genetic testing versus continuing with cholesterol testing” (FM-NAR, S92). Notably, most of the participants who reported wanting more information on cholesterol testing to balance discussion of genetic testing were survey participants. Importantly, these survey participants primarily reported being diagnosed with FH through clinical methods like cholesterol testing and family history, with 32% reporting having had FH genetic testing.

Finally, participants expressed the importance of *reassuring at-risk relatives that there are effective, lifesaving treatments for FH*. Participants stressed that testing was the first step to learning who has FH, which was necessary for potentially getting the right treatment to reduce FH-associated health risks. Ultimately, they described framing the test as “an opportunity”:

[Clinicians] should say is that this is an opportunity to get ahead of the risk factor. When I found out about [my FH], I was in my 60s. I did what I needed to do, but in the long run, it would have been much better if I had known about it when I was in my 30s or 40s. That’s one of the real benefits, because if it doesn’t seem to be a threat right now, people are less likely to do something about it. So, trying to get people to understand that this is an opportunity to avoid some significant distress on your body. (FH-Dx, DI7)

Moreover, participants suggested clinicians offer to lay out a plan if the relative learns they have FH and emphasize optimism as treatment plans can be lifesaving, especially when started early. For instance, this participant suggested saying, “Here’s what we can do. Here are things we can do. Here are our options. Here are our resources” (FH-Dx, DI5). Other participants suggested providing reassurance about potential effective treatments by saying, “Let them know early intervention changes the outcome. That’s the biggest thing” (FH-DX, DI3), and “if [FH] is treated, they can lead normal long lives. They just have to take meds” (FM-AR, DI3). Essentially, they wanted the message to capture “that the medicine helps!” (FH-Dx, S62) and “with treatment you could avoid a heart attack and stroke, possibly” (FH-Dx, DI8). Participants explained that describing FH as treatable helps them maintain hope and face fears:

Letting them know it’s not hopeless … that there’s so many different treatments. … That is the biggest thing. I think it is fear that they’re afraid everything is going to be so dramatically changed at the end of it. (FH-Dx, DI3)

### RQ2 (efficacy): improving self-efficacy

Participants also identified three message features to address self-efficacy (i.e., relatives’ ability to pursue cascade testing), which included breaking down associated barriers. First, participants described how a message feature was needed for *clarifying the cost of cascade testing and what low-cost testing options may be available.* They identified cost as a barrier for their family members and suggested explaining “that genetic testing may not be THAT expensive” (FH-Dx, S1). Participants said that programs for reducing cost could mitigate this barrier, specifically, programs from health systems, advocacy organizations, and genetic testing companies that offer no-cost or low-cost cascade testing for blood relatives of an FH proband or other reduced cost options for cascade testing. However, participants said communication about cost (within and outside of such programs) should be clearer so relatives were not uncertain or “suspicious” of costs (i.e., or concerned “I will receive an unpleasant surprise when a bill arrives” FH-Dx, S2).

Second, participants advocated for *providing step-by-step instructions on how to pursue cascade testing.* This could include what type of clinician to seek testing through, where and how to get genetic or cholesterol testing, and how to get tested if the relative was not a part of the proband’s health care system or lived in a different state. As this participant explained, without clear instructions and steps, participants would have unanswered questions that could inhibit their ability to perform the health protective behavior:

[The letter] mentions a genetic counselor so I’m like, oh so, I have to find a genetic counselor in my area and they will be the one that will order a test or can I just go to my family doctor and they will be the one that will order the test? (FM-AR, DI4)

Finally, participants suggested *providing information and emotional support resources*. Participants recommended offering links to credible information about FH that included contact information so relatives could call a clinician to ask questions directly and a link to the FH Foundation to connect with educational materials and the broader FH community. For example, participants said it may feel overwhelming for relatives to respond to their FH risks without this additional emotional support. This participant explained this, saying:

To think if you got tested for [FH], and then were positive, and were just told, “Okay, figure it out yourself.” No support, nothing like that. That’s terrifying. I think it’s so important to be able to say, “Okay, regardless of testing results, if you have questions or need support, we provide that” (FH-Dx, DI1).

Participants suggested providing different forms of information such as “newsletters” (FH-Dx, S22), “video to share to explain FH” (FH-Dx, S7), and a “list of educational and medically sound resources, like the FH Foundation website that can answer questions and help [them] moving forward” (FM-AR, S97). As this participant noted, “It’s great that resources are being given to us versus us having to try to scramble to find the resources that we need” (FH-Dx, DI6).

## Discussion

This study explored perspectives of individuals and families with FH on how to effectively communicate with at-risk relatives about FH-related health risks and cascade testing, guided by the EPPM [24]. Findings from this study demonstrate the potential importance of eliciting an emotional response like fear to motivate message processing and uptake of a health protective behavior [26]. Participants expressed the need to stress the health threat of FH in clinicians’ messages, otherwise at-risk relatives would see the information about FH as irrelevant and disregard it. Further, the fear appeals participants recommended hinged on communicating about FH as a serious hereditary disorder distinct from ordinary high cholesterol induced by lifestyle factors. Clinicians should take care to recognize and describe this difference as individuals with FH are often misdiagnosed or diagnosed later in life (median age of 47 years old), despite having a family and personal history of persistent high cholesterol [7]. Missed and delayed diagnosis for FH represent a missed opportunity for early and appropriate, risk-reducing medical interventions (e.g., high-intensity statin therapy) [7, 36]. Differentiating FH as a serious genetic condition from ordinary high cholesterol can both attend to severity and stress the susceptibility of other relatives to inheriting the condition.

Participants described susceptibility of family members as an important motivator for cascade testing. They suggested the threat to family could overcome an individual’s complacency and move them to take a family-focused approach to their medical decision making. Previous research supports this kind of messaging, as individuals with FH have reported feeling a moral duty to warn relatives about their FH-related health risks and described wanting to protect relatives from heart disease [13, 37]. Research in hereditary cancer and uncertainty management similarly found family-focused appraisals of risk motivated information-seeking about the condition and decisions to pursue genetic testing [38, 39]. Taking a family-focused approach to communicating about FH may be a particularly effective message design feature, as participants saw the potential to diagnose and treat children as especially motivating.

While eliciting a fear response may be recommended when discussing FH-related health risks, clinicians should also employ message features to bolster at-risk relatives’ efficacy, so they enact a danger control response [25]. Previous research in risk communication testing fear appeals has extensively documented the importance of preventing the “boomerang effect,” in which the fear appeal is so threatening that individuals respond by avoiding information to cope with their fear (i.e., a fear control response) [25, 40]. Incorporating the participants’ suggested response efficacy message features could empower at-risk relatives to pursue cascade testing. Without appropriate diagnosis, individuals with FH may face undertreatment for their high cholesterol and elevated risk for heart disease [1, 7]. Participants also suggested explaining to at-risk relatives that they may not have FH and that genetic testing could also show who did not inherit FH in the family. It may provide relief to at-risk relatives to learn they do not have FH, which could provide additional motivation to pursue cascade testing.

Interestingly, participants’ recommendations for communicating response efficacy included two steps: (a) get tested to get a definitive diagnosis for FH so you can (b) start lifesaving treatment early, if appropriate. Although the health protective behavior of interest in this study is cascade testing, participants recommended clinicians explain to at-risk relatives that if they are diagnosed with FH after cascade testing, clinicians can offer effective treatments and can create a tailored plan for their care. Participants noted that their at-risk relatives may feel overwhelmed without this information and said including information about effective treatments can offer reassurance and an actionable next step, which may ensure a danger control response [25]. Indeed, Hardcastle et al. found individuals with FH believed medications could effectively manage high cholesterol due to FH, which mitigated their anxiety about FH [12]. However, adding this secondary response efficacy message may present challenges in practice as clinicians attempt to communicate about testing and treatment (i.e., potentially two health protective behaviors). Fear and efficacy appeals are most effective for one-time only health behaviors like cascade testing and less effective for repeated health behaviors like adherence to cholesterol-lowering medications [27]. Thus, this type of messaging may be effective for motivating cascade testing, but clinicians may need continued communication and outreach with individuals diagnosed with FH to promote repeated, prolonged adherence to treatments for FH [41].

Findings on message features to improve at-risk relatives’ self-efficacy illustrate the need to reduce barriers that can confound intentions to pursue cascade testing for FH. Effectively communicating about the cost of testing and low-cost programs available to improve access to cascade testing was a prevalent recommendation from participants. It is imperative that clinicians caring for individuals and families with FH are aware of low-cost cascade testing options and clearly communicate about the accessibility of testing. Participants responded positively to programs from health care systems and genetic testing companies offering low-cost genetic testing as overcoming a potential barrier to their relatives’ intentions to test. Such programs may overcome patient-perceived and health care system barriers to paying for cascade testing for FH [37, 42, 43]. Additionally, participants stressed the importance of providing specific steps for the cascade testing process and offering specific resources (like the FH Foundation) for psychosocial support to bolster self-efficacy to pursue cascade testing for FH.

There are several directions for future research building on the current findings. First, future research can statistically test message components of fear and efficacy appeals for FH. Previous research informed by the EPPM has experimentally tested message design to increase/decrease fear and efficacy and measured participants’ feelings of susceptibility, severity, response efficacy, and self-efficacy as well as their message acceptance or rejection and attitudinal and behavioral responses [27]. Similar study design can be used to test the effects of clinicians’ messages informed by this study’s findings and demonstrate which message features and constructs of the EPPM have the strongest predictive effects on intentions and actions to pursue cascade testing among at-risk relatives. Second, communications from clinicians applying these recommendations from individuals and families with FH should be tested in prospective trials. A recent pilot intervention incorporating motivational interviewing and the EPPM in genetic counseling with parents of a child with FH demonstrated increased disclosure and cascade testing rates among at-risk relatives [44]. Findings from the current study complement this pilot genetic counseling intervention by providing examples of message features future interventions can incorporate to communicate about FH risk and motivate cascade testing uptake. Finally, individuals with FH and clinicians have expressed that innovative tools such as chatbots and direct contact of at-risk relatives by clinicians would be helpful ways for clinicians to facilitate family communication [20]. However, future research should investigate how clinicians’ influence may be moderated by the mode of communication used to interact with at-risk relatives. For instance, synchronous communications between clinicians and at-risk relatives may strengthen fear and efficacy appeals as clinicians can more clearly express tone and benefit from the immediacy of those interactions compared to the less rich media formats of written communications [45].

### Limitations

The important contributions of this study must be contextualized within the limitations present. The sample is predominantly Caucasian and reported high educational attainment and household income, which limits generalizability. More diverse families with FH may face meaningful differences accessing health services like cascade testing and recommended care for FH. Without efforts to improve access to health care services for diverse groups and improve efficacy to pursue cascade testing, it may be ineffective to use fear appeals stressing the severity and susceptibility of FH. More diverse families with FH may have additional needs to improve self-efficacy and access to care beyond these participants’ recommendations. Furthermore, participants were recruited through Geisinger’s MyCode and MDLC as well as through the FH Foundation, which may have caused a selection bias for participants who are more active in pursuing testing and information about FH [46]. However, participants described how they and/or their at-risk relatives at times avoid information about FH or put off testing and treatment, suggesting selection bias may not have been as prevalent in this exploratory research.

## Conclusions

Informed by the EPPM, these findings fill an important research and care gap regarding how clinicians can effectively communicate with at-risk relatives by using fear and efficacy appeals that may empower families and motivate cascade testing for FH. However, we are careful not to suggest a “one-size fits all” approach in these recommendations. Clinicians should partner with patients and families to further tailor their communication with at-risk relatives. Given the rates of missed or delayed diagnosis and undertreatment for FH, applying recommendations from individuals and families with FH to clinical communication about FH-related health risks helps address a serious public health gap. These recommendations may also apply more broadly in guiding clinical communications motivating cascade testing for other hereditary conditions.
